# The rate of dasotraline brain entry is slow following intravenous administration

**DOI:** 10.1007/s00213-020-05623-8

**Published:** 2020-08-19

**Authors:** Robert Lew, Cristian C. Constantinescu, Daniel Holden, Richard E. Carson, Vincent Carroll, Gerald Galluppi, Kenneth S. Koblan, Seth C. Hopkins

**Affiliations:** 1 Sunovion Pharmaceuticals Inc., 84 Waterford Dr, Marlborough, MA 01752 USA; 2 Invicro, 60 Temple St, Suite 8A, New Haven, CT 06510 USA; 3 Department of Radiology and Biomedical Imaging, Yale University, P.O. Box 208048, New Haven, CT 06520 USA

**Keywords:** Dopamine transporter, Dopamine, Nonhuman primate, PET, [^18^F]-FE-PE2I, [^11^C]-Raclopride

## Abstract

**Rationale:**

Drugs that rapidly increase dopamine levels have an increased risk of abuse. Dasotraline (DAS) is a dopamine and norepinephrine reuptake inhibitor characterized by slow oral absorption with low potential for abuse. However, it remains unclear whether intravenous (i.v.) administration would facilitate the rapid elevation of dopamine levels associated with stimulant drugs.

**Objective:**

To assess the kinetics of DAS across the blood-brain barrier and time to onset of dopamine transporters (DAT) inhibition.

**Methods:**

We compared the onset of DAT occupancy and the associated elevation of synaptic dopamine levels in rhesus monkey following i.v. administration of DAS or methylphenidate (MPH) using positron emission tomography (PET). Brain entry times were estimated by reductions in [^18^F]-FE-PE2I binding to DAT in rhesus monkeys. Elevations of synaptic dopamine were estimated by reductions in [^11^C]-Raclopride binding to *D*_2_ receptors.

**Results:**

Intravenous administration of DAS (0.1 and 0.2 mg/kg) resulted in striatal DAT occupancies of 54% and 68%, respectively; i.v. administered MPH (0.1 and 0.5 mg/kg) achieved occupancies of 69% and 88% respectively. Brain entry times of DAS (22 and 15 min, respectively) were longer than for MPH (3 and 2 min). Elevations in synaptic dopamine were similar for both DAS and MPH however the time for half-maximal displacement by MPH (*t* = 23 min) was 4-fold more rapid than for DAS (*t* = 88 min).

**Conclusions:**

These results demonstrate that the pharmacodynamics effects of DAS on DAT occupancy and synaptic dopamine levels are more gradual in onset than those of MPH even with i.v. administration that is favored by recreational drug abusers.

## Introduction

The risk of recreational abuse of psychostimulant drugs is associated with drug administration that yields rapid and large increases in synaptic dopamine concentrations in key brain areas, particularly the ventral striatum. Recreational abusers may alter the route of self-administration to achieve more rapid delivery of drugs to the brain to induce multiple “highs.” Drug liking and abuse potential are higher when drugs such as cocaine or heroin are administered intravenously compared with other rapid modes of delivery such as insufflation (Resnick et al. 1977; Comer et al. 1999). Similarly, intravenous methylphenidate (MPH) produces a “high” that is almost indistinguishable from that of intravenous cocaine (Wang et al. 1997) and is not observed when administered orally (Volkow et al. 2002). Drugs that increase synaptic dopamine levels with fast onset kinetics are associated with drug liking and therefore have a greater likelihood of stimulant effects and abuse. Conversely, drugs with a slow onset of effect typically have reduced abuse potential (Busto and Sellers 1986; Farré and Camí 1991; Volkow et al. 1995; Volkow and Swanson 2003).

Dasotraline (1R,4S)-4-(3,4-dichlorophenyl)-1,2,3,4-tetra-hydronaphthalen-1-amine (DAS) is a dopamine and norepinephrine reuptake inhibitor with slow absorption (*t*_max_, 10–12 h) and slow elimination (*t*_1/2_, 47–77 h) pharmacokinetics following oral administration in humans. In a study involving recreational stimulant users, orally administered DAS was found to have a low potential for abuse compared with MPH (Koblan et al. 2016), a finding likely related to the markedly slower oral absorption of DAS relative to MPH (2–3 h to peak). In rat microdialysis studies, DAS produces dose dependent but gradual increases in dopamine efflux in the nucleus accumbens taking up to 2 h to reach maximum effect post-dose, whereas psychostimulants such as d-amphetamine, phentermine, and MPH dose dependently evoked rapid increases in dopamine efflux that peaks at 40 min and declines rapidly thereafter (Rowley et al. 2017).

The slow absorption kinetics for DAS following oral administration reflect a combination of physical properties (e.g., elevated protein binding which leads to reduced free (unbound) drug concentration and high lipophilicity) that might also limit the kinetics of DAS across the blood-brain barrier and slow the onset of dopamine transporter (DAT) inhibition. Here we applied PET imaging of both DAT and dopamine D_2_\D_3_ receptors to assess the kinetics of DAS across the blood-brain barrier and its arrival at the site of pharmacodynamic action for psychostimulant drugs. A series of DAT positron emission tomography (PET) experiments with the radioligand [^18^F]-FE-PE2I at steady state was performed to directly explore the rate of brain entry and binding of both DAS and MPH to DAT. We then performed a series of D_2_\D_3_ receptor imaging with [^11^C]-Raclopride using a 2-day, multiple scan design aimed to determine and compare the timing and elevation of endogenous dopamine caused by blocking of dopamine reuptake with the two drugs.

## Materials and methods

### Radiotracer and drug preparation

DAS (SEP-225289-01 ((1R,4S)-4-(3,4-dichlorophenyl)-1,2,3,4-tetra-hydronaphthalen-1-amine), batch 060-0003) and MPH ((±)-Methyl α-Phenyl-α-(2-piperidyl)acetate hydrochloride, C_14_H_19_NO_2_·HCl, Sigma-Aldrich M2892, batch SLBQ7776V) were formulated as free bases in 100 mM acetate buffer pH 4.5 with 20% hydroxypropyl-β-cyclodextrin and saline, respectively. [^18^F]-FE-PE2I (DAT) and [^11^C]-Raclopride (D_2_\D_3_) were prepared as previously described (Schou et al. 2009; Langer et al. 2000).

### PET studies

#### Animal care and monitoring

[^18^F]-FE-PE2I and [^11^C]-Raclopride PET imaging experiments were carried out under institutional animal care protocols complying with Federal regulations. Animal care approval and oversight for this study was provided by the Yale University Institutional Animal Care and Use Committee.

PET experiments within an animal were spaced at least 2 weeks apart to allow recovery from anesthesia. Animals were fasted for 18–24 h prior to each PET scan experiment. At 2–2.5 h before radiotracer injection, the animal was anesthetized with ketamine (10–15 mg/kg) and glycopyrrolate 0.01 mg/kg i.m., transferred to the PET camera, and intubated with an endotracheal tube for continued anesthesia with 2.0–2.5% isoflurane administered through a rebreathing circuit. An intravenous line was placed and used for injection of radiotracer and administration of the drug. A heated water blanket was used to maintain normal body temperature (37 °C). Vital signs, including heart rate, blood pressure, respiration rate, oxygen saturation, and body temperature, were monitored approximately every 1 to 15 min during the study (data not shown).

#### [^18^F]-FE-PE2I imaging

Two male and two female rhesus macaques (*Macaca mulatta*) housed at the Yale University School of Medicine (New Haven, CT) were used for the [^18^F]-FE-PE2I imaging. In all experiments, [^18^F]-FE-PE2I was administered as a bolus plus constant infusion (*K*bol from 168 to 216 min; total radioactivity = 162 ± 27 MBq) over 4 h to establish steady-state DAT binding. Bolus/infusion delivery parameters (i.e., *K*bol) for [^18^F]-FE-PE2I for each monkey were determined from baseline [^18^F]-FE-PE2I scans that utilized single bolus intravenous injections of the radiotracer (Carson et al. 1993). Identical procedures were used for list-mode image acquisition with a Siemens Focus 220 PET scanner for all DAT brain studies. The dynamic PET imaging series were reconstructed into 57 frames (6 × 0.5 min, 3 × 1 min, 2 × 2 min, and 46 × 5 min) using filtered back projection with standard corrections for random, scatter, and attenuation provided by the camera manufacturer.

At 2 h into the [^18^F]-FE-PE2I scan, under tracer steady-state conditions, DAS or MPH was administered i.v. either as a 3 min i.v. bolus or as a bolus plus 4-step infusion over 40 min (four 10-min steps). The drug bolus experiments were carried out in all four rhesus macaques, each receiving DAS at 0.2 and 0.1 mg/kg and MPH at 0.5 and 0.1 mg/kg. The drug bolus plus 4-step infusion experiments were conducted in two rhesus macaques, each receiving DAS bolus injection over 30 s followed by a 4-step infusion over 40 min (four 10-min steps, 1.55, 1.45, 1.4, 1.3 mg) and MPH bolus injection over 30 s followed by an infusion over 40 min (four 10-min steps, 1.15, 1, 0.85, 0.75 mg).

#### [^11^C]-Raclopride imaging

Two female rhesus macaques (weight, 8.4 ± 0.2 kg; age, 16.6 ± 0.5) were scanned with [^11^C]-Raclopride a total of 8 times, each under 4 scanning conditions per test agent. All scans were performed with [^11^C]-Raclopride (tracer mass limited to 0.25 μg/kg) administered as an intravenous (i.v.) bolus + constant infusion (3 min bolus +132 min infusion; *K*bol = 60 min; injected dose 5.8 ± 1.0 mCi). Scan data collection began simultaneously with tracer injection using the Siemens FOCUS 220 PET scanner. The scan data was acquired for 135 min and binned into sinograms with the following frame timing: 6 × 30 s; 3 × 1 min; 2 × 2 min; and 25 × 5 min. Prior to each tracer injection, a transmission scan was performed with an external gamma source for attenuation correction.

A 2-day design was used to allow for ~ 6 h of consecutive data needed to capture the drug effects over time by combining data from multiple injections of a short half-life (~ 20 min) [^11^C]-Raclopride. Experiment day 1 consisted of an initial [^11^C]-Raclopride injection with MPH given i.v. at 45 min post-start of tracer administration under tracer steady-state conditions (displacement experiment). A second [^11^C]-Raclopride scan was initiated ~ 2.5–3.5 h after the start of scan 1. Experiment day 2 consisted of a MPH pre-block initiated ~ 30 min prior to the first scan. A second scan was again initiated ~ 2.5–3.5 h after the start of scan 1. This same 2-day scanning paradigm was completed for both subjects and repeated using DAS in place of MPH. The test agent doses used were 0.2 mg/kg for DAS and 0.5 mg/kg for MPH. A minimum of 2-week recovery time was allowed between scans with the same subject.

#### Blood sampling

Arterial samples were collected following tracer injection during i.v. bolus baseline PET experiments with [^18^F]-FE-PE2I for measurement of radioactivity concentrations in whole blood and plasma and for measurement of the parent fraction of [^18^F]-FE-PE2I over time. Some samples were used to measure the non-metabolized fraction of tracer and to generate the metabolite corrected arterial input function for quantitative analysis.

Venous blood samples (1 ml) were collected during the respective bolus and 4-step infusion displacement studies to measure the plasma levels of either DAS or MPH. Blood samples were collected in K_3_ EDTA vials, processed for plasma, frozen, and stored at − 20 °C until required for HPLC analysis of either DAS or MPH levels.

#### [^18^F]-FE-PE2I image analysis

Reconstructed dynamic PET images were transferred and analyzed using the image processing software package PMOD 3.6 (PMOD Technologies, Zurich, Switzerland). The PET images were normalized to an MR rhesus brain template (Rohlfing et al. 2012) and a region of interest (ROI) atlas including the caudate nucleus, putamen, and cerebellar cortex was applied. Average activity concentration (kBq/cc) within each ROI was determined and time-activity curves (TAC) representing the regional brain activity concentration over time were generated. TACs were additionally expressed in SUV units (g/ml) by normalizing by the weight of the animal and the injected dose.

Reductions in radiotracer binding following administration of DAS or MPH were used to determine DAT occupancy while reductions in radiotracer binding over time were used to estimate brain entry and binding rates. The displacement half-time is defined as the time it takes from drug administration until 50% of maximal tracer displacement is achieved. Tracer displacement half-times were estimated using average uptake levels from the baseline and displacement experiments and expressing the change in standardized uptake values (SUV) units as a fraction (normalized) of the observed SUV difference at the end of the experiment. Normalized SUV values for caudate and putamen respectively were plotted against time for each dose of DAS and MPH respectively to estimate the drug displacement half-time.

Brain entry of DAS and MPH was determined by analyzing the [^18^F]-FE-PE2I TACs in the putamen and caudate nucleus using a kinetic model using the cerebellar cortex as a reference region. The kinetic model is based on the generalized reference tissue model (GRTM; Votaw et al. 2002) and a recent report of brain entry and binding rates measurement (Nicolas et al. 2016). Here, a one-tissue compartment was used to describe the tissue kinetic, similar to the simplified reference tissue model (SRTM; Lammertsma et al. 1996), with the introduction of a time-varying binding potential post-administration of DAS or MPH at 120 min:

$$ {\mathrm{BP}}_{\mathrm{ND}}(t)={\mathrm{BP}}_{\mathrm{ND}}\left(1-r\left(1-{e}^{-\alpha \left(t-{t}_0\right)}\right)\right), $$

where BP_ND_ is the binding potential prior to displacement, *r* is the maximum DAT occupancy induced by DAS or MPH, *α* is the rate of entry and binding of the drug to the target and *t*_0_ is the time of intravenous administration of DAS or MPH (120 min here). The half-life of the brain entry and binding of the drug was estimated as ln(2)/α. The kinetic model employs 5 parameters: *R*_1_, BP_ND_, *r*, *α*, and *k*_2_′ where *R*_1_ is the relative tracer influx rate constant and *k*_2_′ is the reference tissue tracer efflux rate constant. The maximum occupancy was constrained to the measured displacement, while *k*_2_′ and α were constrained to a common value across the putamen and caudate nucleus. The maximum displacement (*O*^Max^) by DAS\MPH in each brain region was computed as O^Max^(%) = (TAC^Baseline^ − TAC^Displ.^)/TAC^Displ.^x 100, where TAC^Baseline^ is the average TAC values just before DAS or MPH administration (100–120 min p.i.) and TAC^Displ^ is the maximum displacement (225–240 min p.i.), corrected for the non-specific binding (i.e., after subtracting cerebellar cortex values).

#### [^11^C]-Raclopride image analysis

Dynamic scan data were reconstructed with a filtered back-projection algorithm with corrections for attenuation, normalization, scatter, and randoms. ROIs were manually delineated on a single representative anatomical rhesus monkey magnetic resonance image (MRI) registered to a template image. Regions used in this study were caudate and putamen with the cerebellum as the reference region. Registration parameters were obtained to apply the ROIs to individual PET scans, and regional TACs were generated for the ROIs. Apparent binding potential (BP_ND_) curves (target-to-cerebellum ratio - 1) were calculated for putamen and caudate by pooling the data from the 4 injections for each animal/drug combination. The following models were used to describe the apparent BP_ND_ curves. Model 1 is based on a gamma function where the occupancy increases, reaches a maximum, and then decreases, and both phases can be measured in the study. Model 2 has only increasing occupancy with time, approaching an asymptotic value.

- Model 1: Four-parameter model (gamma function):

The change of BP_ND_ over time is modeled using a gamma function.

$$ {\mathrm{BP}}_{\mathrm{ND}}(t)={\mathrm{BP}}_0\left(1-A{\left(t-{t}_0\right)}^{\alpha}\kern0.1em \exp \left(-\beta \left(t-{t}_0\right)\right)\right)\kern0.5em \left(t\ge {t}_0\right) $$

The model can be reformulated as follows:

$$ {\displaystyle \begin{array}{c}{\mathrm{BP}}_{\mathrm{ND}}(t)={\mathrm{BP}}_0\left(1-{\mathrm{Occ}}^{\mathrm{max}}{\left(\frac{t-{t}_0}{t_{\mathrm{max}}-{t}_0}\right)}^{\alpha}\exp \left(\alpha \left\{1-\frac{t-{t}_0}{t_{\mathrm{max}}-{t}_0}\right\}\right)\right)\kern0.24em \left(t\ge {t}_0\right)\\ {}\alpha \equiv -\frac{\ln \kern0.2em 2}{\left(1-\tau \right)+\ln \kern0.1em \tau}\\ {}\tau \equiv \frac{t_{\raisebox{1ex}{$1$}\!\left/ \!\raisebox{-1ex}{$2$}\right.,\mathrm{R}}-{t}_0}{t_{\mathrm{max}}-{t}_0}\kern0.24em \left(\;\tau <1\right)\kern0.6em \mathrm{or}\kern0.36em \frac{t_{\raisebox{1ex}{$1$}\!\left/ \!\raisebox{-1ex}{$2$}\right.,\mathrm{F}}-{t}_0}{t_{\mathrm{max}}-{t}_0}\kern0.24em \left(\;\tau >1\right)\kern11.39999em \end{array}} $$

where *t*_0_ is the known time of drug administration and BP_0_ is the apparent BP_ND_ at time *t*_0_. The maximum occupancy, Occ^max^, is obtained at time, *t*_max_. The occupancy rises to 50% of Occ^max^ at the time, *t*_1/2,R_ (< *t*_max_), and falls to 50% of Occ^max^ at time, *t*_1/2,F_ (> *t*_max_). The 4 parameters of this model are BP_0_, Occ^max^, *t*_max_, and *t*_1/2,R_ (or *t*_1/2,F_).

- Model 2: Three-parameter model:
  $$ B{P}_{\mathrm{ND}}(t)=B{P}_0\left(1- Oc{c}^{\mathrm{max}}\left(1-\exp \left(-\beta \left(t-{t}_0\right)\right)\right)\right),\beta =\frac{\ln \kern0.22em 2}{t_{\raisebox{1ex}{$1$}\!\left/ \!\raisebox{-1ex}{$2,\mathrm{R}$}\right.}-{t}_0}\kern0.24em \;\kern0.24em \;\left(t\ge {t}_0\right) $$

The parameters of this model are BP_0_, Occ^max^, and *t*_1/2,R_.

## Results

Animals tolerated i.v. administration of DAS or MPH at all doses examined. No changes outside physiologically accepted limits in heart rate, respiratory rate, blood pressure, oxygen saturation, and temperature after administration of DAS or MPH compared with baseline levels (data not shown). Transient increases during test agent administration followed by stable elevations in pulse rate and systolic pressure was noted in some cases.

### [^18^F]-FE-PE2I displacement with DAS/MPH

**Fig. 1:**
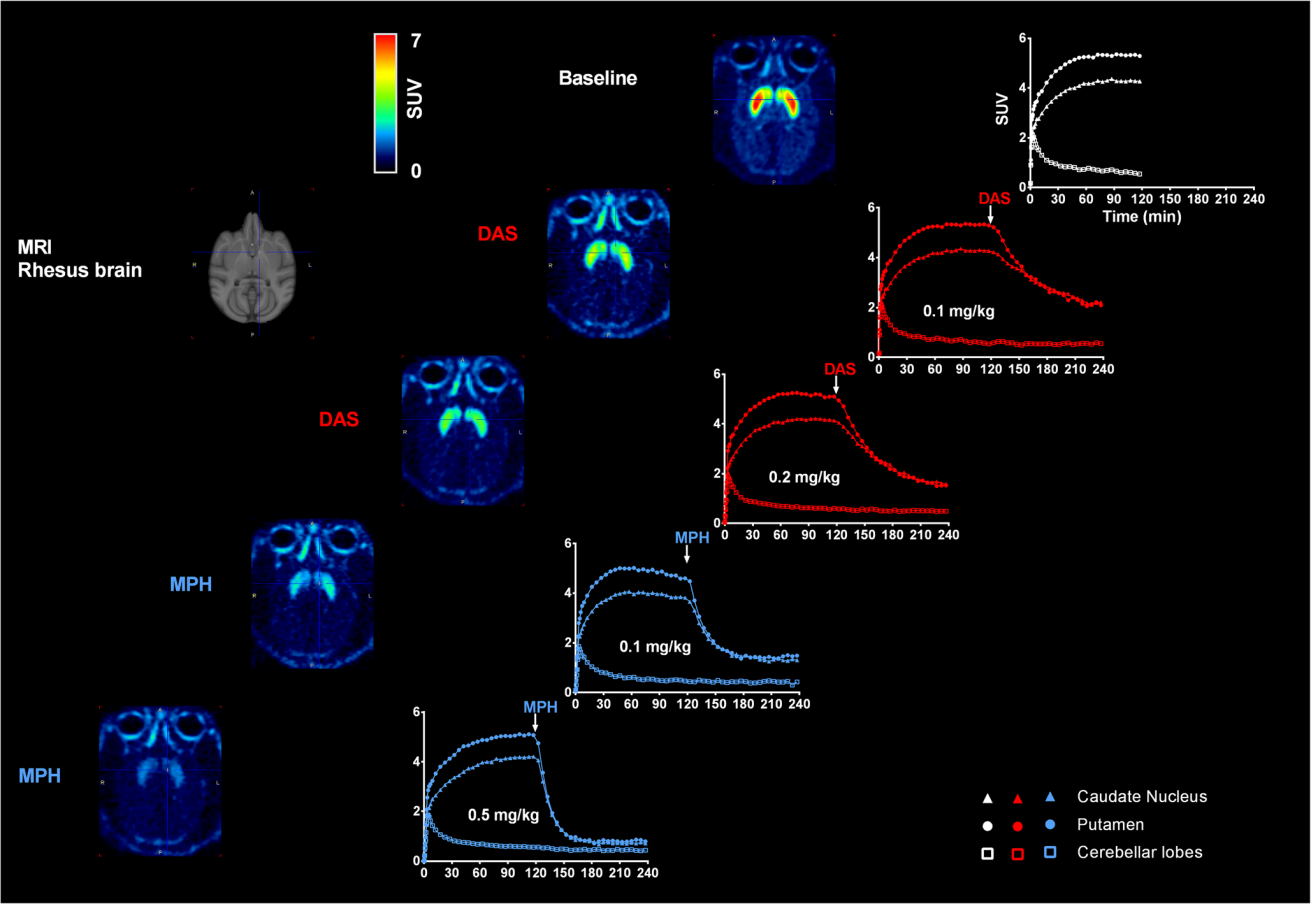
Displacement of [^18^F]-FE-PE2I following intravenous bolus administration of either DAS or MPH in a representative rhesus monkey. DAS (0.1 mg/kg and 0.2 mg/kg) and MPH (0.1 mg/kg and 0.5 mg/kg) were administered intravenously at 120 min after initiation of [^18^F]-FE-PE2I infusion (and scanning). Averaged PET scans (in SUV units) before and after DAS and MPH treatment suggesting a dose-dependent displacement (or occupancy) by DAS and MPH (see also Table 1). Corresponding TACs for caudate nucleus and putamen for DAS (0.1 and 0.2 mg/kg) and MPH (0.1 and 0.5 mg/kg) from 0–240 min suggests a faster displacement of [^18^F]-FE-PE2I by MPH compared with DAS

Figure 1 shows the acquired and averaged PET images (in a representative animal) at baseline (0–120 min) and after administration (120–240 min) of either DAS or MPH and the corresponding TACs for caudate and putamen. Inspection of the PET images clearly demonstrates both drugs to reduce [^18^F]-FE-PE2I binding (i.e., intensity) in a dose-dependent manner and is confirmed by the corresponding TACs for caudate and putamen respectively. The % maximum displacement of [^18^F]-FE-PE2I binding (equivalent to % DAT occupancy) in caudate and putamen by DAS and MPH in each monkey was determined and is summarized in Table 1. Overall, the mean % maximum displacement ± SD of [^18^F]-FE-PE2I binding (or % DAT occupancy) in caudate putamen by 0.1 mg/kg and 0.2 mg/kg DAS was 54.48 ± 10.04% and 68.13 ± 10.30% respectively, while 68.6 ± 5.21 and 88.2 ± 2.62% displacement was observed for 0.1 mg/kg and 0.5 mg/kg MPH respectively.

**Table 1:** Percent maximum displacement (= % DAT occupancy) of DAS and MPH administered as either 3 min bolus or bolus plus 4-step infusion, in the caudate and putamen (plus mean ± SD) of four rhesus primates (A, B, C, D). Maximum plasma levels (*C*_max_) for each monkey for the bolus administrations are provided; averaged plasma levels for each monkey for the infusion period are provided

Drug dose	Animal	*C*_max_ (ng/ml)	Caudate nucleus (%)	Putamen (%)	Caudate\Putamen mean (%)
DAS 0.1 mg/kg	A	87.9	56.1	66.3	61.2
	B	25.9	49.9	55.0	52.5
	C	102	40.8	41.5	41.1
	D	54.6	53.4	72.8	63.1
Mean ± SD		71.9 ± 23.4			54.48 ± 10.04
DAS 0.2 mg/kg	A	190	67.9	77.1	72.5
	B	213	70.1	72	71
	C	123	50.7	55.3	53
	D	NA	64.8	87.3	76
Mean ± SD		175.3 ± 26.9			68.13 ± 10.30
MPH 0.1 mg/kg	A	137	72.4	73.7	73.1
	B	24	72.7	73.5	73.1
	C	177	64.2	62.9	63.6
	D	117	60.4	68.8	64.6
Mean ± SD		169.2 ± 28.4			68.60 ± 5.21
MPH 0.5 mg/kg	A	1130	91.7	90.9	91.3
	B	823	87.9	89.0	88.5
	C	609	83.9	85.9	84.9
	D	729	87.0	89.1	88.1
Mean ± SD		822.7 ± 111			88.20 ± 2.62
Drug dose	Animal	Average plasma (infusion period) (ng/ml)	Caudate nucleus (%)	Putamen (%)	Caudate\putamen mean (%)
DAS 6.0 mg infusion	A	34.86	83.7	87.4	85.6
	B	32.38	82.0	81.3	81.7
Mean		33.62			83.6
MPH 4.3 mg infusion	A	111.9	87.9	89.9	88.9
	B	91.52	90.2	88.9	89.5
Mean		105.7			89.2

*NA*, not available

**Fig. 2:**
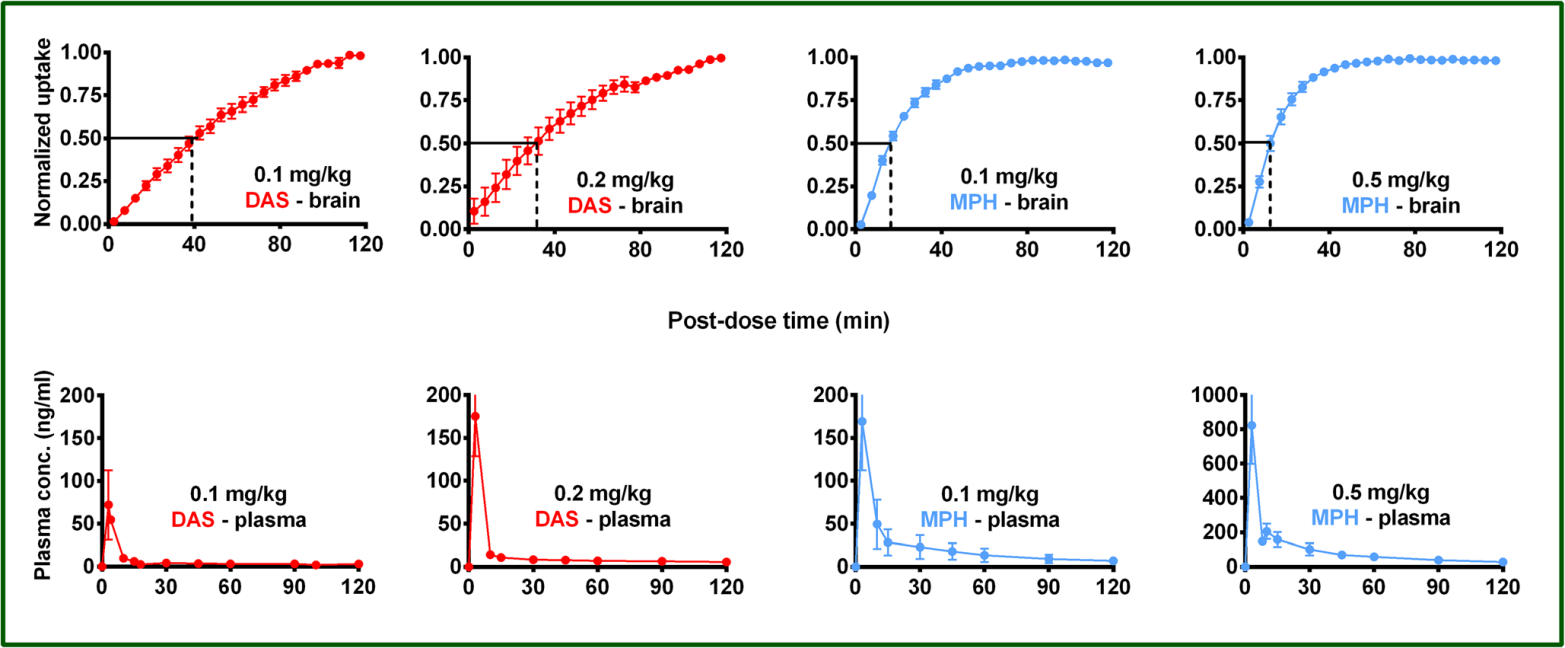
Top row: averaged normalized uptake vs time of DAS (0.1 and 0.2 mg/kg) and MPH (0.1 and 0.5 mg/kg), bottom row: corresponding plasma PK profiles. Averaged displacement half-times between caudate and putamen for MPH (0.1 mg/kg = 15.5 min; 0.5 mg/kg = 11.5 min) was approximately 3-fold faster than that observed for DAS (0.1 mg/kg = 40 min; 0.2 mg/kg = 31 min). The overall plasma PK profile of DAS and MPH was generally similar although Cmax for MPH (0.1 mg/kg: 169.25 ± 28.4 ng/ml; 0.5 mg/kg: 822.7 ± 111 ng/ml) was generally higher than that for DAS (0.1 mg/kg: 71.9 ± 23.4 ng/ml; 0.2 mg/kg: 175.3 ± 26.9 ng/ml)

Figure 2 shows the mean normalized uptake curves for caudate and putamen for each test dose of DAS and MPH. Active uptake of MPH approaches the horizontal asymptote relatively sooner than that for DAS. The averaged displacement half-time (between caudate and putamen) for 0.1 mg/kg and 0.5 mg/kg MPH was 15.5 min and 11.5 min respectively, while for 0.1 mg/kg and 0.2 mg/kg DAS, it was 40 and 31 min respectively. Therefore, MPH displaces [^18^F]-FE-PE2I binding or occupies DAT approximately 3 times faster than DAS

The modified General Reference Tissue Model analysis (Votaw et al. 2002) was employed to estimate brain entry times of DAS and MPH (see Table 2). The mean (± SD, *n* = 4) estimated brain entry times from bolus administrations of DAS were 22.15 ± 5.85 min and 15.32 ± 3.45 min at 0.1 mg/kg and 0.2 mg/kg respectively. For MPH, the mean brain entry time was 3.02 ± 0.65 min at 0.1 mg/kg and 2.14 ± 0.25 min at 0.5 mg/kg, thus demonstrating under similar bolus delivery conditions, MPH enters the brain 4–11 fold faster than DAS.

**Table 2:** Brain entry times (min) of DAS and MPH, administered as 3 min bolus or as a bolus plus 4-step infusion, estimated with modified GRTM applied to putamen and caudate nucleus data in rhesus primates

Animal	Brain region	Bolus administration				Bolus + 4 step infusion	
		0.1 mg/kg DAS	0.2 mg/kg DAS	0.1 mg/kg MPH	0.5 mg/kg MPH	6 mg DAS	4.3 mg MPH
A	Caudate	27.13	19.65	3.41	2.19	16.07	7.15
	Putamen	18.83	17.71	2.59	2.08	16.33	5.77
B	Caudate	25.06	12.09	2.60	1.78	16.34	6.48
	Putamen	33.22	12.60	2.91	2.09	18.57	7.69
C	Caudate	19.23	13.12	3.44	2.65	-	-
	Putamen	19.5	16.03	4.28	2.00	-	-
D	Caudate	18.91	19.90	2.57	2.25	-	-
	Putamen	15.35	11.47	2.34	2.07	-	-
Mean		22.15	15.32	3.02	2.14	16.83	6.77
SD		5.85	3.45	0.65	0.25	1.17	0.83

**Fig. 3:**
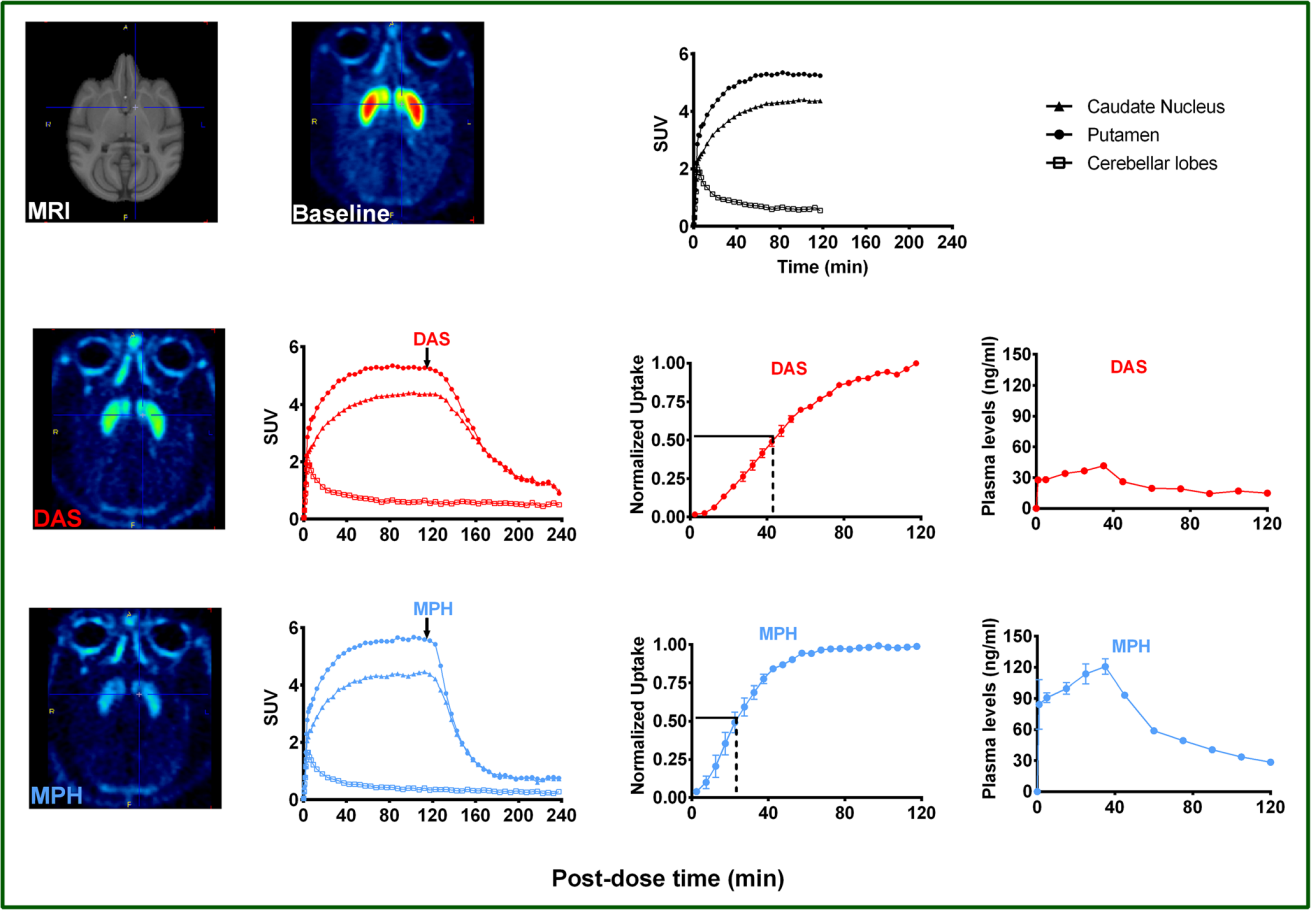
Infusion of DAS or MPH versus infusion of [^18^F]-FE-PE2I in a representative rhesus monkey. DAS (total dose = 6 mg) or MPH (total dose = 4.3 mg) was administered as 30 s bolus + 4 step infusion over 40 min beginning at 120 min after initiation of [^18^F]-FE-PE2I infusion (and scanning). Averaged PET scans (in SUV units) before and after drug treatment are shown to demonstrate a similar reduction of [^18^F]-FE-PE2I intensity (binding) in caudate putamen by DAS (85.6%) and MPH (87.9%; see also Table 1). Corresponding TACs and displacement vs time plots for caudate nucleus and putamen and mean plasma profile (*n* = 2) for DAS and MPH. Even when delivery of drug was matched, MPH exhibited almost a 3-fold faster brain entry than DAS

The plasma pharmacokinetic profile of different doses of DAS and MPH is shown in Fig. 2. Maximum plasma concentration (Cmax) for both doses of MPH (0.1 mg/kg: 169.25 ± 28.4 ng/ml; 0.5 mg/kg: 822.7 ± 111 ng/ml) was generally higher than those for DAS (0.1 mg/kg: 71.9 ± 23.4 ng/ml; 0.2 mg/kg: 175.3 ± 26.9 ng/ml. High plasma drug concentration could be a driving factor (due to the concentration imbalance between the periphery and brain with respect to brain entry of drug and achievement of equilibrium between the brain and the peripheral system. To address this question, monkeys (*n* = 2) were each infused with either DAS (total dose = 6.0 mg) or MPH (total dose = 4.3 mg) over a 40-min period to achieve more comparable initial plasma levels of each drug. Figure 3 shows representative PET scan images before (baseline) and after treatment with either DAS or MPH respectively together with the corresponding TACs and normalized uptake plots in caudate and putamen. Plasma levels for DAS and MPH during (0–40 min post-dose) and after (41–120 min post-dose) the infusion are also shown. Percent maximal displacement of [^18^F]-FE-PE2I by DAS and MPH respectively were comparable (DAS: 81.6 and 85.6%; MPH: 87.9 and 89.5%; see Table 1). TACs and normalized uptake of DAS and MPH in caudate and putamen again show MPH to be faster than DAS in reaching maximum displacement of [^18^F]-FE-PE2I. GRTM analysis of the PET data shows that the brain entry of MPH (= 6.77 min) was faster than DAS (= 16.83 min) by almost 3-fold (Table 2).

### [^11^C]-Raclopride PET studies

[^11^C]-Raclopride D2-PET was used as a non-invasive means of measuring the rise in synaptic DA levels following bolus administration of either DAS or MPH and to further explore the functional significance of the slower displacement/occupancy by DAS with respect to MPH.

**Fig. 4:**
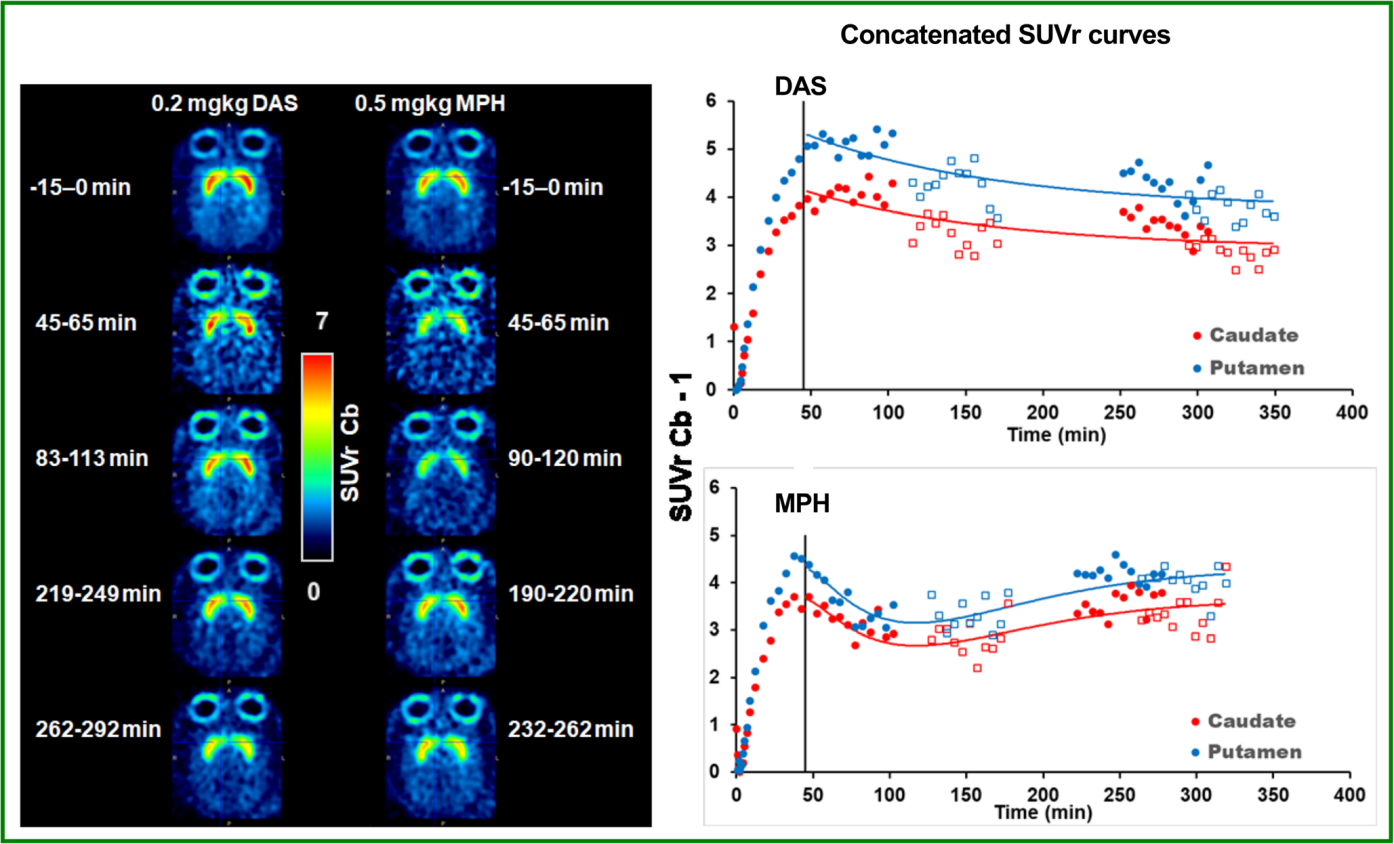
Left panel: from top to bottom, images of SUV ratio to cerebellum (SUVrCb) before and after administration of dasotraline (DAS) and methylphenidate (MPH) as indicated by the time intervals on the left-hand side (relative to DAS administration) and right-hand side (relative to MPH administration). Right panel: apparent *BP*_ND_ data (SUVrCb-1) with model-estimated curves of the putamen (blue symbols) and caudate (red symbols). Different symbols denote different scan days (day 1, closed circles; day 2, open squares) dates

Figure 4 shows images of the averaged [^11^C]-raclopride SUV ratio to the cerebellar cortex (SUVr) before dosing (baseline) and at four selected times after bolus administration of either DAS or MPH in a representative monkey. Inspection of the SUVr images clearly shows how both drugs reduce [^11^C]-raclopride binding (i.e., intensity) followed by a recovery towards baseline levels that depends on the drug: faster for MPH, slower for DAS. Corresponding plots of [^11^C]-raclopride apparent binding potential (SUV ratio – 1) change over time for caudate and putamen are also shown in Fig. 4 together with the model curves of the data. These plots were generating by concatenating apparent BP_ND_ curve segments from all 4 scans over the 2 experimental days. Note that the curves before 45 min post-injection (day 1 2^nd^ scan and day 2 scans) and after 105 min post-injection (all scans) were not displayed since equilibrium is not reached until at least 45 min post-injection and late sampled data were deemed to be too noisy due to the short half-life of ^11^C.

**Fig. 5:**
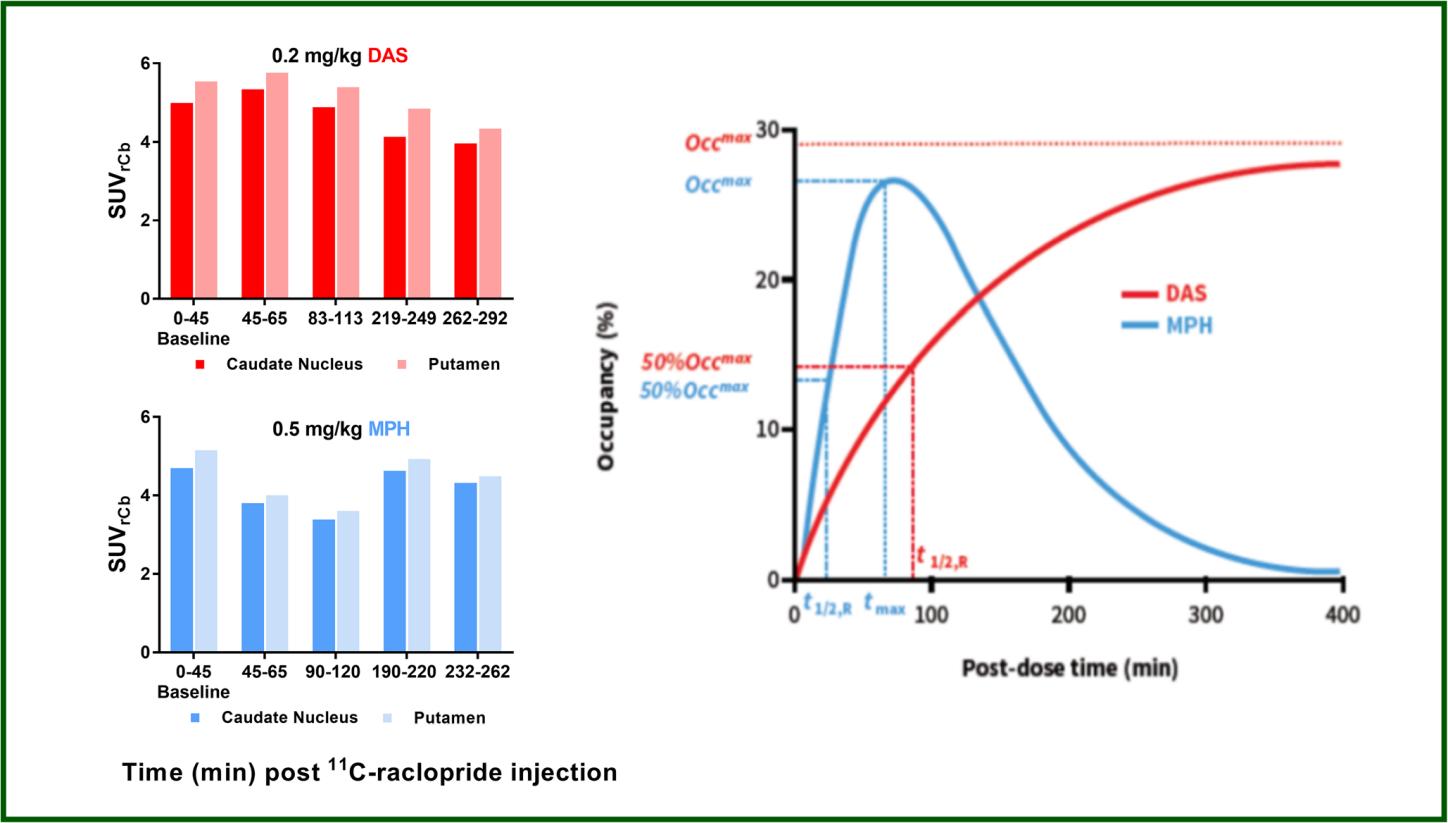
(Left) Average SUV ratio to cerebellum (SUVrCb) at baseline and at 4 times after administration of dasotraline (DAS) 0.2 mg/kg and methylphenidate (MPH) 0.5 mg/kg. (Right) *D*_2_ receptor occupancy curves for MPH and DAS generated using averaged parameters from the two monkeys. Modeling shows the time for half-maximal displacement by MPH was almost 4-fold faster (*t* = 23 min) than that for DAS (*t* = 88 min)

The time course of post-MPH changes was best described by model 1 and that for post-DAS changes by model 2. Based on these model fits, the maximum reductions in [^11^C]-raclopride binding (Occ^max^) in caudate and putamen by MPH (27%) and DAS (29%) were comparable; however, the time for half-maximal displacement (*t*_1/2,R_) by MPH was shorter (*t* = 23 min) by 4-fold than that for DAS (*t* = 88 min), meaning that the elevation of synaptic DA by DAS is slower than MPH. These results compare favorably with the reported brain entry of DAS and MPH. These best data fits using models 1 and 2 are shown in Fig. 5.

## Discussion

The present study assesses the kinetics of DAS and MPH respectively across the blood-brain barrier by measuring the time it takes from drug administration until 50% of maximal tracer displacement is reached. This measurement is the result of both blood-brain barrier (BBB) penetration and rate of binding (association) of DAS (and MPH) to DAT. In regard to DAS penetration of the BBB, in-house in vivo rodent studies with ^14^C-DAS shows higher brain concentrations of DAS relative to plasma (brain:plasma ratio/partition coefficient (*K*_p_) ~ 6.2–12.3; Data-on-file: Sunovion report 360-365). Additionally, in vitro experiments using LLC-PK1 cell monolayers and Xenopus Laevis Oocytes to profile the permeability of DAS indicate it is not a substrate for P-gp nor other efflux transporters of the ATP binding cassette (ABC) superfamily and is not actively taken up by either solute carrier (SLC) and organic anion transporters (OAT) (Data-on-file: Sunovion Reports #531, 544, 575). From these preclinical experiments, it is inferred that DAS penetrates the BBB and enters the brain via passive or transmembrane diffusion.

DAS and MPH were administered intravenously to NHPs in all experiments, therefore avoiding first-pass metabolism (by the liver) and forming metabolites during the measurement period. It should be noted that the major metabolites of DAS (1-keto, 2-hydroxy, and 1-keto DAS) have been shown in a binding panel screen to be of several-fold lower affinity compared with DAS while the major metabolite of MPH (ritalinic acid) is pharmacologically inactive. The current results demonstrate that a slow rate of brain entry of drugs can lead to a marked reduction in psychostimulant properties. The rate of brain entry and DAT occupancy by DAS was slower than that observed with MPH as measured by PET imaging of the rate and degree of displacement of [^18^F]-FE-PE2I from DAT. The slower rate of brain entry and DAT inhibition observed for DAS was associated with a markedly slower rise in synaptic dopamine as measured by the rate and degree of displacement of the radiotracer [^11^C]-Raclopride from dopamine *D*_2_ receptors.

Subjective “highs” in recreational drug abusers correlate strongly with both speed and degree of DAT blockade, with previous research indicating that a short onset time (within 15 min) is required to produce reinforcing effects (Volkow et al. 1997, 2005). In humans, rapid blockade of DAT produces a correlation between the “highs” experienced by individuals and the increases in synaptic DA, as determined by displacement of [^11^C]-Raclopride binding following intravenously administered MPH to healthy subjects (Volkow et al. 1999). Although DAS and MPH bind to DAT sites to produce part of their therapeutic effect, their rate of brain entry and target engagement at DAT sites are substantially different even after intravenous administration. DAS and MPH produced dose-dependent high levels of DAT occupancy in the brains of rhesus monkeys, but the rate of brain entry/DAT occupancy by DAS was ~ 7-fold slower than that of MPH. Moreover, intravenous injection of MPH produced more rapid increases in synaptic DA than DAS, as determined by the displacement of *D*_2_ receptor occupancy by [^11^C]-Raclopride. The time to reach a 50% maximum displacement of [^11^C]-Raclopride binding after DAS administration was ~ 4× longer than after MPH. This direct comparison indicates that the pharmacodynamics of DAS on synaptic dopamine are much slower in onset, more gradual, and more persistent than those of MPH, even following intravenous administration. Based on analogous work by Volkow et al. (1997, 2005) in human subjects, these results suggest that DAS is unable to support the rapid blockade of DAT required to induce the stimulant-like “highs” associated with MPH.

The determination of brain entry was performed using the GRTM model. The GRTM is based on the reference tissue model originally described by Lammertsma et al. (1996). It allows the kinetic rate constant of the PET tracer from the free compartment to the bound compartment to change during the scan to accommodate for direct competition between a tracer and an exogenous ligand, i.e., drug. In assuming that the distribution volume in the target and reference regions are the same and the exchange of drug between bound and free compartments is fast, the GRTM model can fit the observed TACs with a one-tissue compartment model. Thus, following drug administration, the time-varying tracer displacement can be described as

$$ \boldsymbol{O}\left(\mathbf{t}\right)={\boldsymbol{O}}^{\mathbf{Max}}\left(\mathbf{1}-{\mathbf{e}}^{-\alpha \mathbf{t}}\right) $$

where α is the drug delivery rate (effective time for brain entry and effect), with α = ln(2)/T_1/2_ and T_1/2_ is the half-life of displacement associated with the brain entry time. Rapid displacement of [^18^F]-FE-PE2I from DAT and thereby rapid brain entry by intravenous MPH is consistent with previous reported studies in both nonhuman primate and in man (Ding et al. 1997). The time to maximal displacement of [^18^F]-FE-PE2I by MPH is consistent with the rapid euphoric high that occurs with intravenous MPH (Volkow et al. 2005). In contrast, DAS was observed to have slow displacement of [^18^F]-FE-PE2I and slow brain entry (5-11 fold less). The temporal differences in [^18^F]-FE-PE2I displacement by DAS and MPH cannot be attributed to pharmacology since DAS has relatively higher affinity for DAT (IC_50_ = 3 nM; Rowley et al. 2017) than d-threo methylphenidate IC_50_ = 190 nM (Markowitz et al. 2006) and both have been independently reported to have similar DAT occupancies (DAS OC_50_ = 4 ng/ml; DeLorenzo et al. 2011; MPH OC_50_ = 6 ng/ml, Spencer et al. 2006). High affinity DAT compounds generally binds rapidly to DAT with association rates (k_on_) in the order of 1 x 10^6^ M^-1^ s^-1^ while MPH has been reported to have a k_on_ of 8.3 x10^5^M^-1^s^-1^(Hasenhuetl et al. 2015), therefore we assume that both DAS and MPH would bind rapidly to DAT and that the observed time for displacement of the PET tracer is driven mostly by the rate of BBB penetration of the drug.

There are several properties that may affect drug penetration of the BBB including lipid solubility, and protein binding which in turn indicates the unbound or free concentration of the drug presented at the BBB. Regarding lipid solubility, DAS and MPH have cLogP values (measure of lipid solubility) of 4.8 and 2.3 respectively so one would expect DAS to more readily penetrate the BBB than MPH. However, penetration of the BBB is optimal for compounds with LogD (equivalent to Log P for non-ionizable compounds) values between 1-3 (Nicolas et al. 2016) while Pajouhesh & Lenz (2005) has noted that the mean cLogP of marketed CNS molecules to be 2.5. Furthermore, Banks et al (2009) has explained highly lipophilic drugs have lower than expected brain concentrations due to sequestration of the drug into the capillary bed of the BBB and uptake into peripheral tissues, thus lowering the amount of drug concentration that can pass into the brain.

Protein binding of a compound is another property that can influence BBB penetration in that it reflects the unbound or free plasma concentration of drug that can pass through into the brain. Plasma protein binding of MPH is <14% (Faraj et al. 1974) compared to DAS having ~97% plasma protein binding (Data-on-file: Sunovion report 360–455). Consequently, the free plasma concentration of MPH (86%) is almost 30 fold higher than that of DAS (3%) and may partially explain why MPH has a faster brain entry rate than DAS, even when the mean total plasma concentration (Cmax) and DAT occupancy were comparable (see Tables 1 & 2; 0.2 mg/kg DAS Vs 0.1 mg/kg MPH).

While lipid solubility and protein binding influences brain penetration, it appears that it is the combination of these two properties that influences the rate of brain penetration of a drug (Liu et al 2005). Using a physiologically based pharmacokinetic (PBPK) model to describe passive diffusion of drugs into brain, Liu et al (2005) was able to demonstrate that the time to reach equilibrium in the brain was dependent on brain permeability and free drug concentration. The half-time to reach brain equilibrium (t_1/2 equil_) can be estimated by the following equation:

t_1/2 equil_ = V_b_ln2/(PS.f_u,brain_)

where *V*_b_ is brain volume, *PS* is the permeability surface area product, *P* is the permeability rate, *S* is the surface area and *f*_u_ is the unbound (free) drug brain concentration.

For comparative purposes between MPH and DAS, we can assume *V*_b_ and surface area are the same for both drugs, and the above equation becomes:

$$ {t}_{1/2\ \mathrm{equil}}\propto \left[1/\left(P.{f}_{\mathrm{u},\mathrm{brain}}\right)\right] $$

Begley (2004) has compared the permeability rate of 18 compounds of differing lipid solubility (Log P range = − 5 to 5) and demonstrated a positive correlation between permeability and lipid solubility. Extrapolation of these observations to DAS (cLogP = 4.8) and MPH (cLog P = 2.3) suggests that DAS is 12-fold more permeable than MPH. As mentioned above, plasma protein binding of DAS and MPH are ~ 0.97 (or ~ 97%) and ~ 0.14 (or 14%) respectively, and the free plasma concentration of DAS is 0.03 and for MPH, it is 0.86. Since free drug plasma concentration is equal to free drug brain concentration, we can apply the free drug plasma concentration values of DAS and MPH together with the extrapolated permeability for DAS (12P) and MPH (P) to approximate *t*_1/2 equil_ for both drugs, i.e.,

DAS:

$$ {t}_{1/2\ \mathrm{equil}}\propto \left[1/\left(12\mathrm{P}\ast 0.03\right)\right], $$

$$ {t}_{1/2\ \mathrm{equil}}\propto 2.77\mathrm{P} $$

MPH:

$$ {t}_{1/2\ \mathrm{equil}}\propto 1/\left(\mathrm{P}\ast 0.86\right)=1.16\mathrm{P}, $$

$$ {t}_{1/2\ \mathrm{equil}}\propto 1.16\mathrm{P} $$

Liu et al. (2005) have described a low *t*_1/2equil_ value to indicate fast permeability and fast brain equilibrium. Thus, the above demonstrates MPH to be > 2-fold faster than DAS in reaching brain equilibrium and is supportive of the current brain entry results for these two drugs.

A limitation of the current study is that the measured brain entry of MPH and DAS is not reflective of the rapid delivery that is employed amongst drug of abuse users. Due to safety considerations for the test animals, intravenous bolus administration was limited to 3 min. In the real-world setting, bolus i.v. administration of psychostimulants and drugs of abuse among drug abusers is rapid with rates of intravenous delivery occurring from 3–100 s (Samaha and Robinson 2005). It is anticipated that faster brain entry would be observed for both DAS and MPH with a more rapid delivery than that currently employed; however, based on the results with the bolus and infusion experiments, it is unlikely that DAS would attain the same brain entry time or rate of MPH. Another limitation of this study is that it was assumed that 120 min would be adequate time for measuring the displacement of [^18^F]-FE-PE21. While this was true for MPH, it appears DAS was only approaching maximal displacement at 120 min post-dose; therefore, it is feasible that the maximal half-time of DAS may be longer than reported.

For this study, rhesus monkeys were selected as the animal of choice to determine the brain entry rates of DAS and MPH. Aside from PET and PET tracer considerations, rhesus monkeys share 92% genetic homology with humans and their phenotypic similarities extend to almost all aspects of anatomy, physiology, endocrinology, immunology, neurology, behavior, and aging (Mattison and Vaughan 2017). It is likely that the BBB in human and NHP primate to be similar. Also, the cDNA sequence of human DAT and NHP DAT are known and that they share 98.9% homology (Miller et al. 2001). In vitro, metabolic stability experiments in monkey and human liver microsomes and hepatocytes show similar PK profiles in monkey and human (Data-on-file: Sunovion report 360-504). Furthermore, the doses of DAS employed in this study yielded similar plasma levels of DAS that are observed with clinical doses of DAS; therefore, it was expected and confirmed that the DAS RO_50_ from this study (3.5 ng/ml) would be comparable with the reported human RO_50_ values (4.5–6 ng/ml; DeLorenzo et al. 2011, Hopkins et al. 2017). The comparable DAT RO_50_ values for rhesus monkey and human also indicates that the anesthesia used in these experiments did not influence the presented results. Overall, we believe that the observed slow brain entry rate of DAS in rhesus monkey translates across to human.

In summary, intravenously administered DAS enters the brain 5–11-fold slower than MPH. The slow brain entry of DAS leads to a 4-fold slower elevation of synaptic DA as compared with the rapid synaptic DA elevation observed with MPH. We conclude that IV administered DAS is unlikely to support the rapid increase in synaptic dopamine responsible for the abuse liability of stimulants like MPH.

This work was previously presented as a poster communication at the American Professional Society of ADHD and Related Disorders meeting January 2017 “Dasotraline enters the brain more slowly than methylphenidate in rhesus monkeys” R Lew, CC Constantinescu, V Caroll, O Barret, KS Koblan and SC Hopkins.”
